# Unveiling the next generation of MRI contrast agents: current insights and perspectives on ferumoxytol-enhanced MRI

**DOI:** 10.1093/nsr/nwae057

**Published:** 2024-02-07

**Authors:** Guangxiang Si, Yue Du, Peng Tang, Gao Ma, Zhaochen Jia, Xiaoyue Zhou, Dan Mu, Yan Shen, Yi Lu, Yu Mao, Chuan Chen, Yan Li, Ning Gu

**Affiliations:** Jiangsu Key Laboratory for Biomaterials and Devices, School of Biological Science and Medical Engineering, Southeast University, Nanjing 210009, China; Key Laboratory for Bio-Electromagnetic Environment and Advanced Medical Theranostics, School of Biomedical Engineering and Informatics, Nanjing Medical University, Nanjing 210029, China; Key Laboratory for Bio-Electromagnetic Environment and Advanced Medical Theranostics, School of Biomedical Engineering and Informatics, Nanjing Medical University, Nanjing 210029, China; Department of Radiology, the First Affiliated Hospital of Nanjing Medical University, Nanjing 210029, China; Jiangsu Key Laboratory for Biomaterials and Devices, School of Biological Science and Medical Engineering, Southeast University, Nanjing 210009, China; MR Collaboration, Siemens Healthineers Ltd., Shanghai 200126, China; Department of Radiology, Affiliated Nanjing Drum Tower Hospital of Nanjing University Medical School, Nanjing 210008, China; Key Laboratory for Bio-Electromagnetic Environment and Advanced Medical Theranostics, School of Biomedical Engineering and Informatics, Nanjing Medical University, Nanjing 210029, China; School of Mathematical Sciences, Capital Normal University, Beijing 100048, China; Nanjing Key Laboratory for Cardiovascular Information and Health Engineering Medicine, Institute of Clinical Medicine, Nanjing Drum Tower Hospital, Medical School, Nanjing University, Nanjing 210093, China; Jiangsu Key Laboratory for Biomaterials and Devices, School of Biological Science and Medical Engineering, Southeast University, Nanjing 210009, China; Jiangsu Key Laboratory for Biomaterials and Devices, School of Biological Science and Medical Engineering, Southeast University, Nanjing 210009, China; Nanjing Key Laboratory for Cardiovascular Information and Health Engineering Medicine, Institute of Clinical Medicine, Nanjing Drum Tower Hospital, Medical School, Nanjing University, Nanjing 210093, China; Jiangsu Key Laboratory for Biomaterials and Devices, School of Biological Science and Medical Engineering, Southeast University, Nanjing 210009, China

**Keywords:** ferumoxytol, contrast agent, contrast-enhanced MRI, ferumoxytol-enhanced MRI

## Abstract

Contrast-enhanced magnetic resonance imaging (CE-MRI) is a pivotal tool for global disease diagnosis and management. Since its clinical availability in 2009, the off-label use of ferumoxytol for ferumoxytol-enhanced MRI (FE-MRI) has significantly reshaped CE-MRI practices. Unlike MRI that is enhanced by gadolinium-based contrast agents, FE-MRI offers advantages such as reduced contrast agent dosage, extended imaging windows, no nephrotoxicity, higher MRI time efficiency and the capability for molecular imaging. As a leading superparamagnetic iron oxide contrast agent, ferumoxytol is heralded as the next generation of contrast agents. This review delineates the pivotal clinical applications and inherent technical superiority of FE-MRI, providing an avant-garde medical-engineering interdisciplinary lens, thus bridging the gap between clinical demands and engineering innovations. Concurrently, we spotlight the emerging imaging themes and new technical breakthroughs. Lastly, we share our own insights on the potential trajectory of FE-MRI, shedding light on its future within the medical imaging realm.

## Teaser

This review explores how ferumoxytol-enhanced MRI revolutionizes diagnostics with safer contrast options, offering a glimpse into advanced imaging techniques and promising new directions in medical research.

## INTRODUCTION

In the realm of clinical diagnostics, magnetic resonance imaging (MRI) is a pivotal and commonly utilized technique, distinguished by its non-ionizing radiation and non-invasive approach, and its reliance on the enhanced sensitivity afforded by contrast agents (CAs). Generally, the origin of MRI signals is the precession of hydrogen protons within the body, from magnetization by an external field, and can be influenced by longitudinal (T_1_) and transverse (T_2_ and T_2_*) relaxation times. The contrast in MRI primarily stems from the variations in hydrogen proton densities, and the T_1_, T_2_/T_2_* values across different biological tissues. However, the absence of significant differences in relaxation times between pathological and healthy tissues in many scenarios underscores the necessity of employing MRI CAs.

CAs function by modulating the T_1_ and T_2_/T_2_* relaxation times of nearby hydrogen protons, thereby generating new or amplified contrasts among different tissues in MRI. Since the introduction of Magnevist®, the inaugural gadolinium-based CA (GBCA), into clinical application in 1988, there have been extensive efforts to promote the diagnostic precision and applicability of contrast-enhanced MRI (CE-MRI) across various medical indications [1]. In the current landscape, CE-MRI has evolved into a standard tool for disease diagnosis and management on a global scale, with nearly 40% of MRI examinations demanding the usage of CAs for optimal outcomes [2].

MRI CAs authorized for clinical intravenous application are principally divided into three categories, based on their core metallic elements: GBCAs, iron-based CAs and manganese-based CAs (Table 1). GBCAs, being the inaugural class of MRI CAs to gain clinical approval, persist as the predominant choice in clinical MRI practices. The deployment of GBCAs can produce prominent enhancement to amplify the contrast between pathological and normal tissues in MRI, while maintaining an overall satisfactory safety record over a decade. However, the issues of immediate adverse reactions, unique challenges during pregnancy and lactation, and gadolinium toxicity are persistently associated with the use of GBCAs [3]. Furthermore, the association of nephrogenic systemic fibrosis with GBCAs and the accumulation of gadolinium in the human brain were ascertained in 2006 and 2015, respectively [4,5]. These revelations triggered an enduring pursuit for alternative MRI CAs.

**Table 1.** Clinically approved intravenous MRI contrast agents.

Contrast agent category	Trade name	Generic name	Distribution type in body	Indications	Current market status
**Gadolinium-based contrast agents**	Dotarem, Clariscan	gadoterate meglumine		CNS^a^	Marketed
	ProHance	gadoteridol		CNS	Marketed
	Gadovist, Gadavist	gadobutrol		CNS	Marketed
	Magnevist	gadopentetate dimeglumine	Extracellular fluid agent	CNS	Discontinued (2018)
	Omniscan	gadodiamide		CNS	Discontinued (2023)
	Optimark	gadoversetamide		CNS	Marketed​
	Multihance	gadobenate dimeglumine		CNS	Marketed​
	Vasovist, Ablavar	gadofosveset trisodium	Blood pool agent	MRA^b^	Discontinued (2017)
	Primovist, Eovist	gadoxetate disodium, gadoxetic acid	Targeted/organ-specific agents	Liver MRI^c^	Marketed​
**Iron-based contrast agents**	FerahemeFeridex, EndoremResovist	ferumoxytolferumoxidesferucarbotran	Blood pool agent, targeted/organ-specific agentsTargeted/organ-specific agentsTargeted/organ-specific agents	IDA^d^, MRI (off-label)Liver MRILiver MRI	Marketed​Discontinued (2008)Discontinued (2009)
**Manganese-based contrast agents**	Teslascan	mangafodipir trisodium	Targeted/organ-specific agents	Liver and pancreas MRI	Discontinued (2012)

^a^CNS, central nervous system; ^b^MRA, magnetic resonance angiography; ^c^MRI, magnetic resonance imaging; ^d^IDA, iron deficiency anemia.

The combined advantages of superior safety and remarkable imaging effectiveness have broadly validated the CA potential of superparamagnetic iron oxide (SPIO). Within this category, ferumoxytol, a leading SPIO, holds the unique position as the only iron-based CA approved for clinical intravenous use. Initially sanctioned for intravenous iron replacement therapy, ferumoxytol received approval in the USA in 2009, Canada in 2011 and Europe in 2012 for treating iron deficiency anemia in adults with chronic kidney disease (CKD), or those who are intolerant to oral iron or have not responded adequately to it [6]. Although the foray into intravenous iron supplementation therapy may have been a tactical move by the manufacturer, ferumoxytol was, in fact, initially developed as a blood pool CA for MRI [7]. Over the last decade, the distinctive pharmacokinetics and ability to shorten T_1_ and T_2_/T_2_* relaxation times have led to the increasing off-label diagnostic application of ferumoxytol as an MRI CA, exploring extensive utilization in clinical research. Moreover, the recent global discontinuation of Omniscan (gadodiamide), one of the GBCAs, combined with the ongoing phase III clinical trials of another iron-based CA, Ferrotran (ferumoxtran-10), has notably intensified interest in this clinically successful iron-based CA. A hepatocyte-specific CA featuring a manganese ferrite core has been innovatively developed, facilitating faster, more sensitive and higher-resolution hepatobiliary MRI compared to the clinically used gadoxetate disodium, thus positioning it as a promising candidate for the next generation of liver-specific CAs [8]. This development has fostered keen anticipation of expanding our understanding of its current uses and future potential in MRI.

As the landscape of clinical studies on ferumoxytol-enhanced MRI (FE-MRI) continues to expand, several important themes have evolved. For example, after the Food and Drug Administration (FDA)’s recommendation of slow titration in 2015 [9], quick dosing of ferumoxytol became less common. Over time, as more clinical data on MRI contrast use in the pregnant and pediatric populations have come to light, concerns about the safety of FE-MRI have lessened. Concurrently, the incorporation of FE-MRI across various domains, coupled with an increased understanding of its unique advantages, has spurred research areas such as arteriovenous separation, multimodal image fusion and micrometer-scale imaging of tiny brain vessels.

This review has three primary objectives. First, we delve into the current clinical indications of FE-MRI, spotlighting the distinctive attributes that distinguish ferumoxytol from GBCAs. To offer a comprehensive understanding, we focus on the continuity and cohort size of various clinical indications, thereby highlighting the core areas of FE-MRI research. We provide an online Excel sheet as supplementary material for this. Second, we delve into the emerging themes within FE-MRI, particularly its convergence with advancements in computer science. This interplay bridges comprehension gaps in nanomedicine and within the medical and engineering communities regarding FE-MRI. Our discussion includes cutting-edge developments in brain microvascular imaging, rapid imaging methodologies and advanced multimodal image fusion and segmentation techniques. Finally, drawing on our experience with ferumoxytol synthesis and insights from ongoing clinical trials, we share our perspective on the promising trajectory of FE-MRI applications.

## FERUMOXYTOL’S CHARACTERISTICS IN MRI

### Distinctive characteristics and versatility of ferumoxytol in MRI

Structurally, ferumoxytol is composed of an iron oxide nanoparticle enveloped by a polydextrose sorbitol carboxymethyl ether coating. This unique structure, with a mean colloidal particle size of 30 nm and a molecular weight of 731 kDa, imparts distinctive CA properties and versatile characteristics [10].

**Figure 1.**
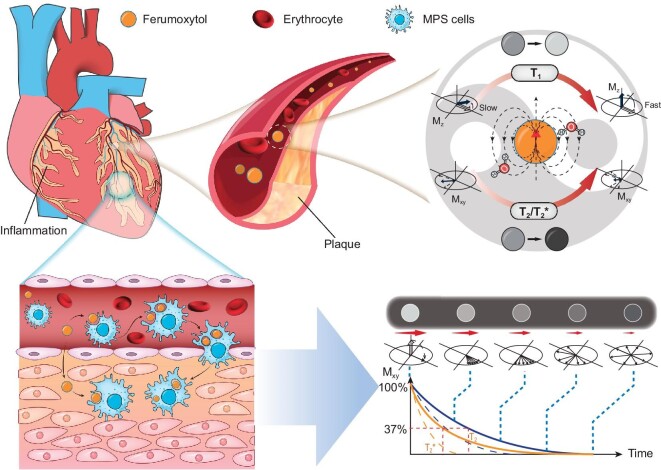
Schematic diagram of the principles of FE-MRI. During the blood pool phase, it significantly shortens the T_1_ and T_2_ relaxation times of the blood, thus achieving bright-blood effects in T_1_-weighted imaging and dark blood effects in T_2_/T_2_*-weighted imaging. In the delay phase, by the local aggregation of ferumoxytol disrupting the uniformity of the local magnetic field, the decrease of the transverse magnetization vector M_xy_ is expedited, resulting in a negative contrast effect.

**Figure 2.**
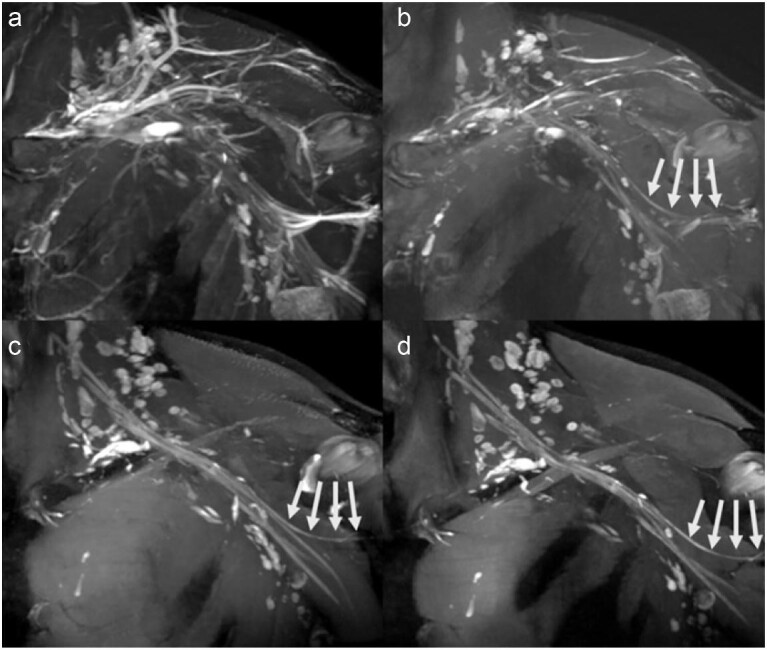
FE-MRA exhibits a black-blood effect in the 3D T_2_-weighted sequence, which improves the visibility of the axillary nerve (marked by white arrows). (a and c) Flow suppression turned off. (b and d) Flow suppression turned on. Reproduced with permission from ref. [14]. Copyright 2022, Radiological Society of North America (RSNA).

Firstly, in comparison to GBCAs, ferumoxytol exhibits higher longitudinal relaxivity (*r*1) and transverse relaxivity (*r*2), enabling its use as both T_1_ contrast (positive contrast) and T_2_ contrast (negative contrast). Particularly at lower magnetic field intensities, the benefit of decreasing the T_1_ relaxation time is further amplified [11]. For instance, at 1.5 T, the *r*1 relaxivity for ferumoxytol in a biological medium is 19.0 mM^−1^ s^−1^, while the *r*2 relaxivity is 64.9 mM^−1^ s^−1^ [12]. In comparison, GBCAs exhibit values of 3.6–6.3 and 4.3–8.7 mM^−1^ s^−1^, respectively [11]. This characteristic enables ferumoxytol-enhanced magnetic resonance angiography (FE-MRA) to be utilized for both bright-blood and black-blood imaging (Fig. 1), enhancing the accuracy of diagnoses. For instance, FE-MRA is capable of depicting deep venous thrombosis of the lower extremities, and is able to illustrate the extent of the thrombus even in the case of slow flow [13]. Furthermore, the suppression of minor venous blood signals through black-blood imaging can enhance nerve visualization, leading to increased diagnostic confidence [14] (Fig. 2).

Secondly, ferumoxytol's prolonged blood pool phase and delayed intracellular uptake make it highly suitable for multimodal imaging across various vascular beds. Its significant particle size ensures long retention in the blood pool, preventing substantial leakages from the vascular bed, and it can bypass kidney clearance [15]. Conventional CAs, GBCAs, can permeate from the vascular bed into the extracellular fluid swiftly, resulting in a brief intravascular retention time ranging between 2 and 11 minutes [16]. The abbreviated intravascular half-life presents challenges for the practicing radiologist in choosing the right imaging time frame, especially when imaging the venous system. In contrast, ferumoxytol has an intravascular half-life of around 14.5 hours at a concentration of 4.0 mg/kg [17]. It aids in executing complex and time-consuming sequences, enabling high-resolution imaging of whole-body multivessel territories. This facilitates sub-millimeter resolution imaging of the cardiovascular system under electrocardiographic gating [18], capturing intricate vascular structures with complex hemodynamics like arteriovenous malformations (AVMs) [19], permitting repeat imaging without extra contrast material [20]. These attributes are particularly beneficial for pediatric patients with smaller cardiovascular dimensions, patients with multiple vascular lesions, or those who have difficulty following breath-holding commands, potentially reducing the need for anesthesia. In the delayed phase, the assessment of inflammation and the imaging of the mononuclear phagocyte system (MPS) are readily attainable [21]. In short, FE-MRI empowers the ongoing monitoring of structural and functional anomalies on a system-wide level, including the evaluation of vascular access before and after organ transplantation, as well as tracking inflammation changes before and after treatment.

Finally, ferumoxytol stands out as a multifunctional formulation. It not only expedites FE-MRI acquisition but also simultaneously serves as an iron supplement for neonates with congenital heart disease (CHD). This is particularly advantageous since most neonates in the intensive care unit are found to be iron-deficient [22]. Moreover, because of the inherent magnetic properties, nanoenzymatic activities and immunomodulatory effects of ferumoxytol, this agent has spurred an array of innovative preclinical therapeutic explorations. These explorations span tumor treatments, immune regulation in sepsis, antimicrobial and anti-inflammatory treatments for dental caries and periodontitis, tissue engineering, and magnetic nerve stimulation aimed at alleviating depression and myocardial infarction, among others [23].

### Biological distribution, metabolism and safety overview of ferumoxytol

A distinguishing feature of ferumoxytol compared to GBCAs is its three-phase post-injection progression, which accommodates diverse imaging applications: the dynamic phase, the blood pool phase and the delayed phase.

After the bolus injection of ferumoxytol, the dynamic phase unfolds promptly, capturing the initial passage of ferumoxytol through the vasculature. Its defining characteristic is the rapid and dynamic distribution of ferumoxytol within the vascular system. During the dynamic phase, the primary application is dynamic perfusion imaging, used for assessing alterations in tissue perfusion efficiency resulting from hemodynamic changes or completing separate imaging of arteries. Despite the current recommendation by the FDA for intravenous drip administration of ferumoxytol to mitigate the risk of serious allergic reactions, some institutions still opt for rapid injection to facilitate dynamic susceptibility contrast (DSC) MR perfusion imaging [7]. As of now, there is no evidence to suggest that small rapid doses can trigger severe allergic reactions. Typically, a ferumoxytol injection undergoes a 4–6-fold dilution to preclude adverse reactions and susceptibility artifacts associated with high concentrations during rapid injection.

Following the dynamic phase, the blood pool phase typically ensues within a few minutes to several dozen minutes after injection and persists for extended durations, often spanning dozens of hours when the dosage is ≥3.0 mg/kg. During this stage, ferumoxytol continues to circulate in the bloodstream but achieves a uniform distribution, ensuring that an adequate concentration of ferumoxytol is retained within the blood vessels. The main imaging methods in this phase include high-resolution T_1_-weighted and T_2_/T_2_*-weighted imaging, leveraging ferumoxytol's distinct characteristic of a sustained vascular presence [17]. Consequently, this phase proves useful for analyzing microvascular structures or perfusion shifts without being influenced by changes owing to vascular permeability. This feature has been exploited for imaging brain microvessels [24].

While MPS cells’ gradual phagocytosis of ferumoxytol begins during the blood pool phase, the delayed phase is characterized by the period extending from dozens of hours to days after injection [25]. It is during this time that ferumoxytol accumulates to concentrations detectable by MRI in MPS-related organs or inflamed tissues. In this phase, T_2_/T_2_*-weighted imaging is most commonly used, and it can also be employed to monitor *in vivo* cell labeling and tracking [26]. Ultimately, ferumoxytol is stored in the liver, spleen and bone marrow, participating in regular iron metabolic activities like red blood cell production and iron supplementation [27].

Ensuring the safety of nanoparticles in biological systems remains a key hurdle for the broader adaptation of nanomedicine [28]. Unlike GBCAs, ferumoxytol is not metabolized by the kidneys and thus does not present a risk of nephrogenic systemic fibrosis or nephrotoxicity, nor are there concerns about intracranial deposition of ferumoxytol. The largest safety reports provide encouraging data on the off-label use of ferumoxytol as an MRI CA. Ahmad *et al.* analyzed 39 studies up to April 2020, involving 4336 patients receiving 5411 doses of ferumoxytol. Their findings revealed a low 2% incidence of adverse events, without any instances of immediate serious adverse events [29]. Ferumoxytol's outstanding safety profile can be attributed to two primary factors. First, iron plays a fundamental role in the standard metabolism of living organisms. Second, the doses of ferumoxytol used for MRI contrast, typically ranging from 3.0–7.5 mg/kg, are substantially lower than those employed for intravenous iron supplementation, which often exceed 510 mg.

In certain groups such as pregnant women and children, extra care is needed when considering the safety of CAs. FE-MRI has been established as safe for children and young adults [30]. Interestingly, ferumoxytol has been applied in iron supplementation for women in their second and third trimesters, even at doses higher than those used for MRI CAs (up to 1020 mg for iron supplementation) [31]. In summary, ferumoxytol is well-suited for a wide range of populations except for patients with iron overload.

## CLINICAL APPLICATIONS DURING THE BLOOD POOL PHASE

As early as 2003 [32], the research on FE-MRI using ferumoxytol has continuously progressed in both the medical profession and academia. In particular, studies by Sigovan *et al.* in 2012 and Bashir *et al.* in 2013, on imaging dialysis fistulae and renal transplant vascularity with ferumoxytol, ignited researchers’ enthusiasm for exploring the potential of FE-MRA in visualizing tubular lumens [33,34]. It is apparent why ferumoxytol has great advantages over GBCAs in the CKD population, such as dual-modal imaging, extended intravascular half-life and absence of nephrotoxicity.

In the realm of MRI CAs, ferumoxytol currently stands as the sole clinically available blood pool agent. Gadofosveset, its only competitor and one of the GBCAs, was withdrawn from the European market in 2009 and the American market in 2017, owing to its weak market sales [35]. Since then, there has been a market void for MR blood pool CAs. The rising off-label applications of FE-MRA have the potential to fill this market void.

In general, the biological distribution and properties of ferumoxytol differ significantly from those of GBCAs, suggesting a range of appropriate indications. Next, we will discuss the advancements in employing FE-MRA for the vascular system, segmented by anatomical regions. It is worth emphasizing that even though FE-MRA indications are delineated based on specific vascular beds, patients can benefit from sequential scans of multiple vascular beds. Once the injection is administered, most safety concerns are largely mitigated.

### Arterial system

#### Peripheral artery disease

Peripheral arterial disease (PAD) severely affects patients’ quality of life, with a reported prevalence of up to 14% in the general population. There is a clear link between low renal function and higher incidence rates. Over the past two decades, GBCA-enhanced MRA (GE-MRA) has emerged as a leading modality for peripheral vascular imaging [36]. Despite its prominence, GE-MRA faces several constraints: (i) it is difficult to precisely control the imaging time, especially in patients experiencing significant hemodynamic changes; (ii) large-field imaging protocols are complex and highly dependent on experience; some institutions use multiple contrast injections to complete multistation imaging [37]; and (iii) the nephrotoxicity associated with GBCAs. Ferumoxytol stands out as an apt blood pool CA to overcome these challenges. Specifically, FE-MRA overcomes the limitations of first-pass imaging and allows for large-field steady-state acquisition. FE-MRA's resolution can attain sub-millimeter precision, paving the way for meticulous stenosis evaluations.

The efficacy of FE-MRA in diagnosing PAD is well-established. Research has demonstrated the efficacy of employing both first-pass and steady-state FE-MRA for assessing peripheral arteries, even in patients exhibiting complex vascular anomalies such as venous malformations [38]. Multiple blinded vascular surgeons were able to make informed clinical decisions based on FE-MRA images [39], highlighting FE-MRA's potential as a viable alternative to computed tomography angiography (CTA) or GE-MRA. FE-MRA allows an expansive field of view, covering the abdominal aorta to the foot, and maintains a high resolution during steady-state imaging [40]. Moreover, superior image quality has been observed in PAD patients with more intricate lesions, including multiple stenoses, entrapments and occlusions [7]. A concern in peripheral vascular imaging is the overlapping of arteries and veins, which might obscure observations. Nonetheless, with high spatial resolutions, distinguishing between arteries and veins becomes feasible through post-imaging processing [41].

#### Transcatheter aortic valve replacement

Transcatheter aortic valve replacement (TAVR) has become a widely adopted treatment option for patients with severe aortic stenosis. According to the American College of Cardiology Expert Consensus, more than 50 000 patients have undergone TAVR in the USA alone since its first approval by the US FDA in 2011 [42]. The chief purpose of TAVR imaging is to accurately portray the structure of the aortic root. Currently, CTA stands as the benchmark for preoperative TAVR planning in aortic stenosis patients [7]. However, widespread renal complications among elderly TAVR patients limit the use of both CTA and GE-MRA [43].

FE-MRA has been demonstrated to be a safe and effective alternative to CTA in patients with renal dysfunction, providing reliable vascular landmarks of the aortic root and peripheral access for TAVR planning [44]. Using respiratory and electrocardiographic gating, FE-MRA can evaluate the ascending aorta within 10 minutes, yielding reproducible measurements of the aortic annulus area [45]. In the largest studies to date, involving 31 patients, TAVR guided by FE-MRA demonstrated a 96% success rate, with no complications observed in any patient within a month after the TAVR [46]. In TAVR procedures of accessing iliofemoral arteries, the intimal diameter and curvature extent are both crucial for predicting the feasibility [7]. Hence, an additional benefit of FE-MRA is the capability of scanning an extra vascular bed for finalizing the surgical route assessment without the need for additional contrast injections. It is notable, especially for those with a smaller iliofemoral minimal lesion dimension.

#### Pulmonary artery and pulmonary embolism

Globally, pulmonary embolism (PE) ranks third among the causes of acute cardiovascular fatalities [47]. Thus, it is imperative to develop a prompt and precise method for diagnosing PE. Computed tomography pulmonary angiography is currently regarded as the imaging standard for the diagnosis of PE [48]. However, there is an urgent need to develop a safer and more accessible examination method for those patients who are concerned about ionizing radiation or have contraindications/allergies to iodinated CAs.

In fact, the ability of FE-MRA to visualize pulmonary vasculature was demonstrated as early as 2003 when Prince *et al.* first used ferumoxytol for cardiovascular MRI [32]. In 2015, the feasibility of using FE-MRA to visualize pulmonary in the pediatric population was reported [49]. In 2019, leveraging the ultrashort echo time sequence with ferumoxytol, Knobloch *et al.* achieved better image quality than GE-MRA in imaging pulmonary vascular, capturing both vascular and non-vascular structures [50].

**Figure 3.**
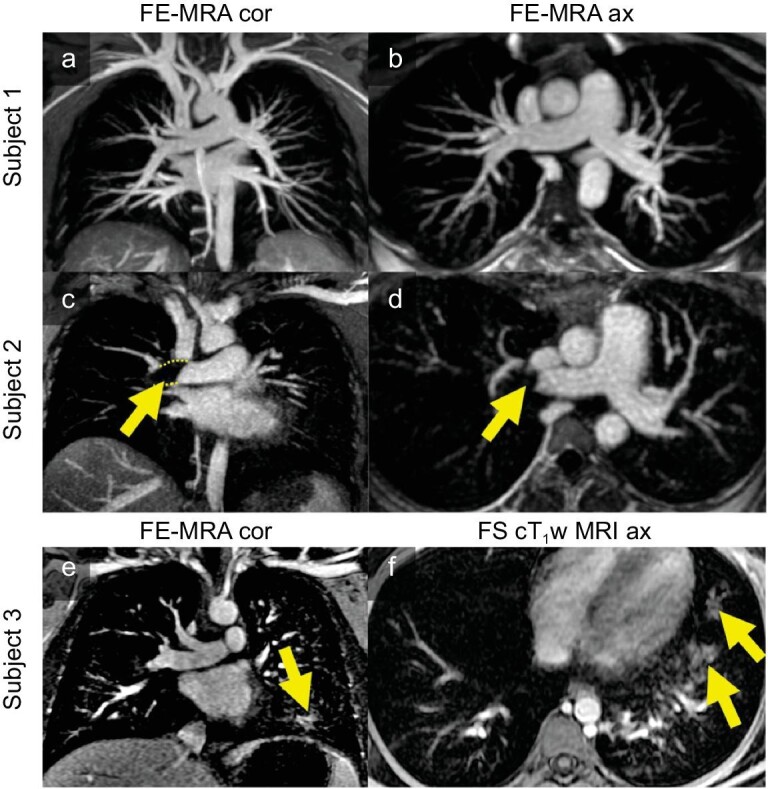
FE-MRA successfully detected pulmonary embolisms in pregnant patients and provided additional findings. Subject 1 is normal, Subject 2 has a detected pulmonary embolism (indicated by arrows) and Subject 3 has detected pneumonia (indicated by arrows). Reproduced with permission from ref. [52]. Copyright 2022, Wiley.

To date, only three studies have emerged that focus on using FE-MRA for PE diagnosis. In 2015, Hope *et al.* presented images illustrating the efficacy of FE-MRA in diagnosing PE, with the location of the embolism clearly identified in two cases [40]. In 2020, Aghayev *et al.* employed FE-MRA and confirmed its use for diagnosing PE in a patient post-kidney-transplantation [51]. In 2022, Starekova *et al.* reported the first case of a pregnant patient with PE diagnosed using FE-MRA [52] (Fig. 3). Despite ongoing advancements highlighting FE-MRA's potential in PE diagnosis, a thorough evaluation of its diagnostic efficacy for PE is essential.

#### Relative cerebral blood volume and functional MRI

Relative cerebral blood volume (rCBV) represents the vessel density in a region of interest and can identify lesions that might be overlooked on anatomical MRI. Typically, DSC MRI is employed to rapidly capture the T_2_*-weighted signal of GBCAs and calculate rCBV. However, its variability leads to inconsistent rCBV thresholds across institutions when distinguishing between high and low vascular distributions. FE-MRA provides more accurate rCBV measurements with reduced dosage, given its unique pharmacokinetics. Specifically, 1.0 mg/kg (≈75 mg Fe) of ferumoxytol yields a DSC MRI enhancement comparable to the standard dose of gadoteridol (>1000 mg Gd) used in DSC MRI [53].

Various methods based on ferumoxytol measurement of rCBV have been reported, primarily for the diagnosis of pseudoprogressive tumors and fine vessels. In a study by Nasseri *et al.*, 48.2% of patients with glioblastoma multiforme were diagnosed with pseudoprogression, of which 30% manifested 3 months post-chemoradiotherapy [54]. These results also prompted a reconsideration of the 3-month time limit for diagnosing pseudoprogression as per the Response Assessment in Neuro-Oncology criteria. Furthermore, this confirms the prognostic value of rCBV assessment using ferumoxytol within a short span of months. The high-resolution rCBV maps, obtained via the steady-state method, are not constrained by acquisition time and can yield a sufficient signal-to-noise ratio to visualize fine vascular structures—something that is challenging in classical low-resolution DSC MRI acquisitions [24]. Fine vascular assessment is important for understanding disease mechanisms and identifying early pathological changes.

The use of ferumoxytol-based rCBV measurement in functional MRI (fMRI) has led to some unique applications. Compared to standard blood-oxygen-level-dependent (BOLD) fMRI, ferumoxytol-enhanced functional blood volume imaging not only presents better spatial specificity but also produces a significant increase in absolute % signal enhancement, being two to three times greater [55]. These advantages arise from the dominant ferumoxytol-enhanced resting-state fMRI signal changes that reflect CBV during neuronal metabolic activity. In contrast, BOLD fMRI relies on the hemodynamic response to neuronal activity as a result of changes in CBV, cerebral blood flow and cerebral blood oxygenation cascades following neuronal activity. This distinction holds particular significance in clinical practice. As the signal originates from both oxygenated arteries and relatively deoxygenated veins, ferumoxytol-enhanced quantitative BOLD fMRI provides a more precise measurement of blood volume fraction in areas like the hypoxic regions of brain tumors [56].

#### Congenital heart disease and coronary MRA

CHD affects an estimated 1% of live births, posing a significant health challenge during childhood [57]. The merits of using MRI in pediatric CHD are well-established. However, cardiac MRI (CMR) in children with CHD is highly specialized and individualized, requiring lengthy examination times and interactive expert supervision, limiting its widespread use [58].

**Figure 4.**
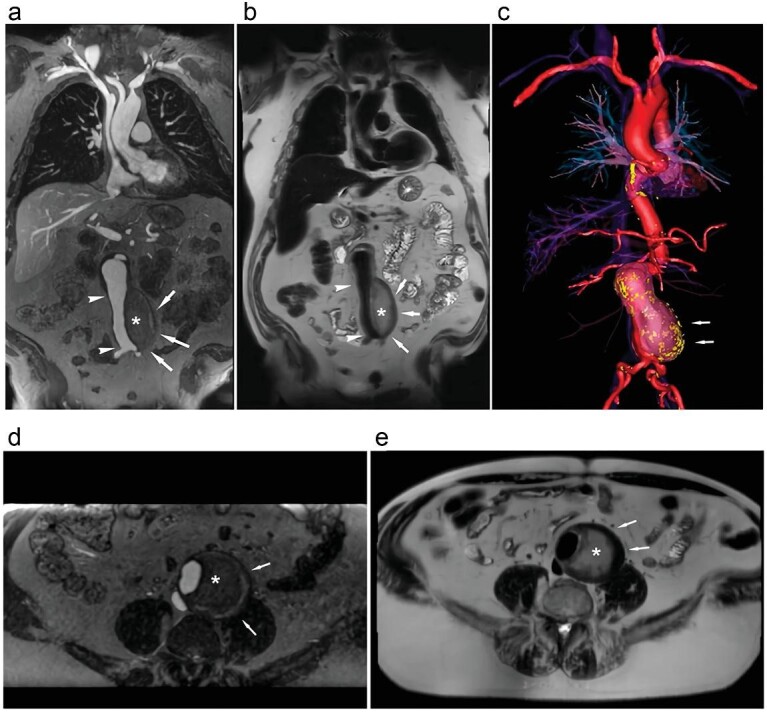
Ferumoxytol-enhanced bright-blood and black-blood imaging of a 71-year-old male post-arterial stent repair. Coronal and axial bright-blood imaging (a and d) and black-blood imaging (b and e) depict the stent graft (white arrow) with no endoleak. Black-blood imaging indicates the formation of a thrombus outside the intima within the aneurysm sac (asterisks, b and e). (c) The 3D color volume rendering illustrates the relationship between the thrombosed aneurysm sac (white arrow) and the vascular intima. Reproduced with permission from ref. [61].

The introduction of ferumoxytol has transformed the landscape of CMR applications for children with CHD, simplifying workflow and heightening productivity. Finn and Hu’s team pioneered and persistently refined a 4D, multiphase, steady-state contrast-enhanced imaging (MUSIC) technique that has shown excellent diagnostic performance and clinical impact in multicenter pediatric CHD patients [18,59]. By integrating parallel imaging and compressed sensing, the 4D MUSIC sequence facilitates comprehensive data capture within 5 minutes [60]. Ferumoxytol-enhanced half-Fourier single-shot turbo spin-echo black-blood imaging is a simple and reliable way to adequately suppress blood signals [61], benefiting from the excellent bimodal imaging capabilities of ferumoxytol (Fig. 4). Black-blood imaging plays a crucial role in characterizing the abnormalities formed on the vessel walls. This innovative technique not only avoids the need for magnetization preparation pulses but also overcomes the flow reliance of traditional black-blood imaging. When paired with the previously mentioned bright-blood imaging, ferumoxytol-enhanced black-blood imaging is exceptionally apt for evaluating endoleaks and thrombi.

Another use-case of FE-MRA worth noting in refining clinical CMR routines is ferumoxytol-enhanced 4D flow MRI, which integrates functional assessment with anatomical visualization. Compared to GBCAs, ferumoxytol-enhanced 4D flow MRI provides higher accuracy in measuring the flow, superior image contrast and effective ventricular mass evaluation [62]. Owing to its extended duration within the bloodstream, ferumoxytol-enhanced CMR lessens the need for patients to undergo anesthesia and withhold breathing [63]. In fact, ferumoxytol-enhanced CMR is viable for scrutinizing intricate CHD in neonates and infants without relying on anesthesia, which is a large step towards curbing anesthetic neurotoxicity risks in the young [64]. Furthermore, both FE-MRA and ferumoxytol-enhanced 4D flow MRI can be undertaken in tandem seamlessly. In the future, boosted by increments of clinical experience and advances in accelerating the imaging speed, ferumoxytol is likely to facilitate 4D flow MRI in large systemic vascular beds throughout the body, especially for cardiac hemodynamic imaging.

**Figure 5.**
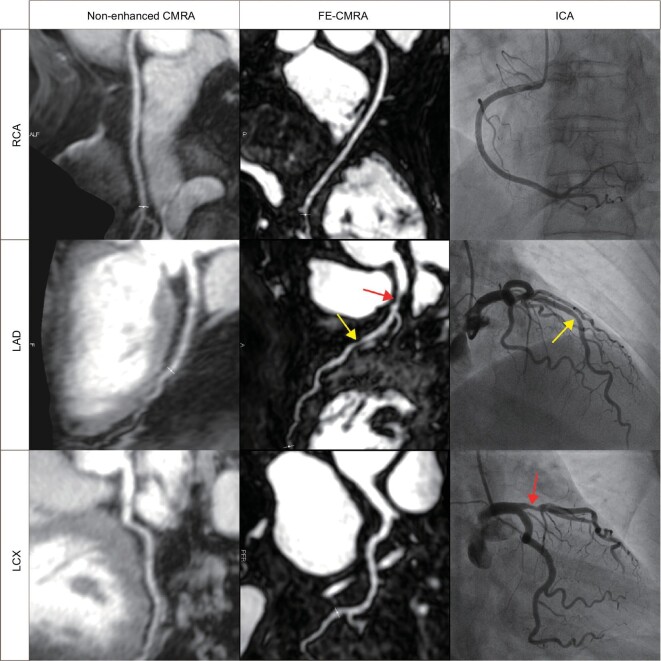
Compared to the gold standard ICA, FE-CMRA successfully diagnosed severe stenosis in the proximal part of the LAD (indicated by the red arrow) and moderate stenosis in the mid-segment (indicated by the yellow arrow). FE-CMRA = ferumoxytol-enhanced coronary magnetic resonance angiography. ICA = invasive coronary angiography. RCA = right coronary artery. LAD = left anterior descending coronary artery. LCX = left circumflex coronary artery. Reproduced with permission from ref. [66]. Copyright 2023, American Heart Association, Inc.

Studies assessing coronary stenosis using FE-MRA have been limited to small samples. Detailed reports on the diagnostic performance of FE-MRA for coronary angiography in larger samples are currently lacking, representing an urgent area in need of investigation. In 2021, Chin and his team first showcased the promising accuracy of FE-MRA when diagnosing 13 individuals with advanced CKD (stage 4–5), achieving a sensitivity of 91.4% and specificity of 94.5% compared to the benchmark invasive coronary angiography [65]. In a recent study, our team investigated 30 patients with suspected coronary artery disease. Using invasive coronary angiography as a reference, we demonstrated the good diagnostic performance of ferumoxytol-enhanced coronary MRA [66] (Fig. 5). This implies that ferumoxytol-enhanced coronary MRA might emerge as a viable alternative to CTA for diagnosing coronary artery disease. The integration of novel acquisition and reconstruction modalities might further promote the use of ferumoxytol-enhanced coronary MRA.

### Venous system

#### Deep venous thrombosis and venous evaluation in pediatric patients

**Figure 6.**
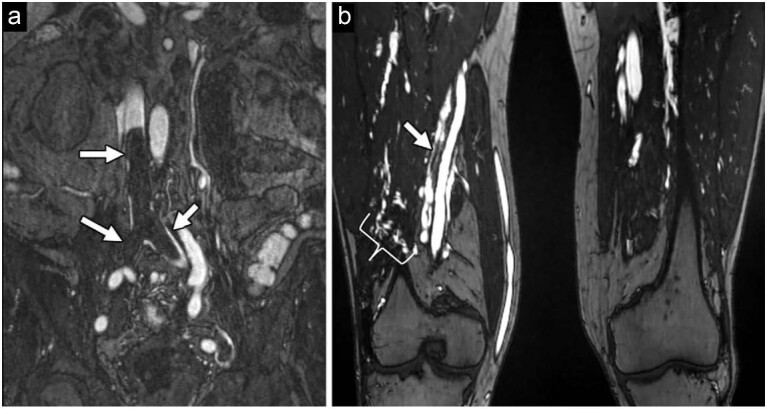
FE-MRA used for deep vein thrombosis detection and classification. (a) The bright signal void on the coronal FE-MRA suggests acute deep vein thrombosis extending from the inferior vena cava to the common iliac vein (arrow). (b) Filling defects in the lower limb FE-MRA, along with the presence of collateral vessels (bracket), indicate chronic deep vein thrombosis. Note the clear differentiation of collateral vessels by FE-MRA. Displaying such collateral vessels might be challenging for GE-MRA. Reproduced with permission from ref. [67]. Copyright 2022, RSNA.

Deep venous thrombosis is a common disease that has potentially serious consequences. Ultrasound examination alone does not offer a reliable assessment of the deep venous system. Assessing deep venous thrombosis using ferumoxytol-enhanced magnetic resonance venography (FE-MRV) is advantageous, primarily because of its effective enhancement of the venous system [67] (Fig. 6). In fact, FE-MRV has shown pronounced venous enhancement across both abdominopelvic and lower limb deep venous systems [68].

In children with CKD, FE-MRV's benefits are accentuated, avoiding contrast nephrotoxicity and radiation. High-resolution FE-MRV has proven to be a reliable diagnostic technique for children with CKD. It offers the advantage of providing multivessel bed vein dissection in a single session [69]. Compared to gadofosveset—the only now-retired gadolinium-based blood pool CA—ferumoxytol provides more durable and consistent enhancement throughout the venous circulation in children [70]. The extended time window of FE-MRV makes it an almost ideal high-resolution venography method for children with various complex anatomies.

#### Preoperative analysis of deep inferior epigastric artery perforator flap

For patients preparing for breast reconstruction using the deep inferior epigastric artery perforator flap, a detailed analysis of the preoperative venous anatomy is imperative. This is especially pertinent as ∼2% of those undergoing this procedure experience venous congestion leading to flap failure. Furthermore, there are added clinical benefits to evaluating the superficial venous systems, especially when deep venous drainage is inadequate. FE-MRV successfully delineated the superficial venous system in every patient [71], in contrast to the GE-MRA examination where the superficial inferior epigastric vein remained unidentified in 15% of the patients [72]. Additionally, FE-MRV may also aid in identifying which flaps require venous augmentation using the superficial inferior epigastric vein [71].

#### Central venous stenosis and occlusion

For patients with end-stage renal disease, central venous stenosis and occlusion present common and frustrating challenges in their treatment [73]. Although several imaging assessment tools are available, each has inherent drawbacks that limit their accuracy in clinically assessing central venous anatomy. Ferumoxytol-enhanced cardiovascular magnetic resonance venography (FE-CMRV) is a new alternative method. In a study by Shahrouki *et al.*, FE-CMRV was successfully acquired for all 52 patients, achieving an almost ideal overall accuracy of 100.0% when compared to catheter venography [74]. In another study using FE-CMRV to assess the diagnostic performance of central thoracic vein stenosis or occlusion, a comparable performance of FE-CMRV was demonstrated, with a sensitivity and specificity of 99% and 98%, respectively, in diagnosing central thoracic vein stenosis or occlusion in 35 patients [75]. This underscores the potential of 3D FE-CMRV as a practical, accurate and robust technique for high-resolution imaging of central veins, which can then guide therapeutic interventions.

### Combined arteriovenous evaluation

#### Arteriovenous fistula surgery planning and routine monitoring

Arteriovenous fistulas (AVFs), the preferred vascular access method for end-stage renal disease patients, exhibit a creation failure rate of 30%–70% and are susceptible to failure during use [76]. Accurate evaluation of vascular access before and after AVF placement is vital for surgical planning as well as determining an AVF’s functional status. MRA is crucial for AVF surgical planning and for pinpointing the causes of AVF failure, given its provision of three-dimensional vascular data that aid in vascular alignment and facilitate evaluations by vascular surgeons. However, GE-MRA is not adequate for more routine monitoring needs [33].

In the evaluation of AVFs, FE-MRA outperforms duplex ultrasound and time-of-flight MRA. Compared to time-of-flight MRA, FE-MRA more consistently provides high image quality for hemodialysis fistula imaging [33]. In contrast to duplex ultrasound, FE-MRA exhibits improved predictive capabilities for successful fistula outcomes (with an odds ratio of 6.5) and was able to identify small arteries that were not suitable for fistula creation (odds ratio, 4.6) [77]. Interestingly, by combining FE-MRA with computational fluid dynamics, one can obtain consistent hemodynamic characteristics both before and after the creation of an AVF. This is achieved by simulating hemodynamics using a realistic vascular model of the patient [76]. These findings have substantial significance for refining surgical blueprints and tailoring patient-centric treatments.

#### Arteriovenous malformations

AVMs arise from anomalies in vascular development during embryogenesis, predisposing patients to ischemia and numerous risks that may exacerbate over time. FE-MRA is predominantly used for diagnosing cerebral AVMs in pediatric patients. Children are more prone to AVM ruptures than adults; hence, AVM management in children deserves particular consideration [78]. The potential of ferumoxytol-enhanced susceptibility-weighted imaging (FE-SWI) for diagnosing various types of vascular malformations was initially demonstrated in 2011 [79]. Overall, FE-MRA can improve the robustness of depicting pediatric AVMs, with a diagnostic performance comparable to CTA and digital subtraction angiography in AVM assessment and detection of residual or recurrent AVMs after treatment, without the risk of radiation [19,78].

FE-MRA also performs well in the diagnosis of pulmonary AVMs for patients with hereditary hemorrhagic telangiectasia. It can detect all AVMs of 2 mm or larger, even in the presence of embolic coils. This is one advantage of FE-MRA in the detection of pulmonary AVMs [80]. Two additional foreseeable advantages of FE-MRA are its capability to simultaneously assess AVMs in the lung, brain and liver using a single ferumoxytol injection, and its potential as a therapeutic approach for anemia. In fact, all hereditary hemorrhagic telangiectasia patients in the authors’ study cohort were anemic, highlighting the significant benefit of using FE-MRA.

#### Allograft vasculature assessment

Vascular complications after solid organ transplantation are common. Addressing the vascular complications of grafts can be complex. This underscores the necessity of a comprehensive understanding of vascular anatomy to ensure effective management of transplanted patients [81].

Compared to digital subtraction angiography, the current gold standard for vascular diagnosis, FE-MRA stands out owing to its non-invasive nature, non-nephrotoxic properties and the ability to simultaneously detail the anatomy of various arteries and veins. Relative to digital subtraction angiography, FE-MRA offers comparable high sensitivity, specificity and accuracy (95.2%–97.6%) for determining the severity of stenosis in transplanted renal arteries, suggesting its potential role as a non-invasive test method to reduce the number of unnecessary routine angiograms [82]. This approach may yield additional benefits in the clinical practice for providing safer dosing outside the scanner and reducing the imaging time, thereby improving MRI examination throughput efficiency. The department of radiology at Duke University Medical Center has adopted FE-MRA as the MR technique of choice for the diagnosis of transplanted renal vasculature if ultrasound is not able to form a valid diagnosis [83]. Apart from being more attractive for radiologists, high spatial resolution vascular images are more intuitive for vascular surgeons to use for comprehensive surgical planning.

## CLINICAL APPLICATIONS DURING THE DELAYED PHASE

In the distinctive delayed phase of ferumoxytol, macrophages break down its carbohydrate shell, allowing the remaining iron oxide particles to enter the MPS. Macrophages play a pivotal role in the inflammatory response, while MPS cells are distributed throughout the body, including in the liver, bone marrow and lymphatic tissue. Therefore, imaging during the delayed phase offers opportunities for non-invasive diagnostic, prognostic and pharmacological evaluations of inflammation, MPS, tumors and cell implants.

From a technical perspective, MRI in this phase achieves a negative contrast effect mainly by T_2_ or T_2_* weighting. The negative contrast can be attributed to a local inhomogeneous magnetic field induced by the phagocytosis of ferumoxytol, leading to further reduced T_2_* relaxation time in the tissue. Since the uptake of ferumoxytol by macrophages is a slow process, delayed enhancement MRI is often performed hours to days after the injection. With just a single injection, patients can benefit from extensive scans of multiple organs, such as systemic atherosclerotic plaque and lymph node (LN) metastasis assessments, based on clinical need.

### Inflammatory imaging

#### Vascular system inflammation imaging

Inflammation plays a crucial role in the pathogenesis of vascular system pathologies. The diagnostic applications of FE-MRI in vascular inflammation cover intracranial aneurysms (IAs), brain AVMs (bAVMs), atherosclerotic plaques and osteomyelitis.

IAs and bAVMs are the leading causes of cerebrovascular lesions, and inflammatory factors significantly contribute to the development and progression of IAs and bAVMs [84]. Unlike conventional methods that focus on anatomical structures, FE-MRI mainly depicts macrophage activity and inflammatory responses at the disease site, crucial for predicting the rupture risk of IAs and bAVMs. In 2012, Hasan *et al.* showed that the early macrophage uptake of ferumoxytol through FE-MRI can prognosticate the rupture risk of IAs, heralding new avenues for non-invasive differentiation of aneurysm stability [85]. Similarly, FE-MRI has been employed to gauge the extent of the inflammatory response in bAVMs, hinting at ferumoxytol's promising potential as an inflammatory response biomarker in bAVMs [86].

Ferumoxytol is also effective for assessing plaque inflammation due to its selective uptake by atherosclerotic plaques. In turn, inflammation in atherosclerotic plaques is an important determinant of plaque vulnerability. So far, FE-MRI has demonstrated its capability as a non-invasive marker for inflammation in the carotid and aortic vessel walls during the delayed phase. Compared to the normal vessel wall, plaque presents a significantly lower signal in delayed-period T_2_*-weighted MR images. Interestingly, in the study by Smits *et al.*, there was no correlation between the uptake in FE-MRI and fluorine-18 fluorodeoxyglucose positron emission tomography (PET)/CT examinations of carotid plaques, which may imply that ferumoxytol shows different pathophysiological aspects of plaque inflammation [87]. Its diagnostic efficacy and clinical value are expected to be further elucidated in larger cohort studies in the future.

Osteomyelitis is an inflammatory disease of the bones and bone marrow that typically originates from hematogenous or traumatic sources. Conventional imaging techniques currently used for diagnosing osteomyelitis have limitations in terms of accuracy and specificity. Utilizing FE-MRI for osteomyelitis imaging has demonstrated its ability to accurately diagnose osteomyelitis, positioning it as a viable alternative to the current standard of care, GBCA-enhanced MRI (GE-MRI) [88]. It is important to note that while FE-MRI primarily provides information on inflammation, GE-MRI mainly focuses on edema. The synergy of information from both approaches may help further improve the specificity of osteomyelitis diagnosis. Regarding diagnostic accuracy, FE-MRI emerges as a promising and potentially more reliable method for diagnosing osteomyelitis.

#### Cardiac inflammation imaging

FE-MRI has been explored for cardiac inflammation imaging in several clinical indications, such as myocardial infarction, myocarditis and cardiac transplantation. FE-MRI is a unique tool capable of assessing cardiac inflammation at the cellular level, which has a significant impact on clinical practice. Ferumoxytol-enhanced multi-parametric CMR characterizes myocardial infarction pathology in more detail, and macrophage infiltration into, and accumulation within, the infarcted region are potential therapeutic targets [89]. FE-MRI can also detect areas of infarction during acute myocardial infarction or the first two weeks after infarction, indicating promising applications for inflammation-level monitoring, risk prediction and efficacy assessment [90]. However, unexpected results were seen in patients with acute myocarditis, in which no significant signal changes were observed in FE-MRI within the myocardium, suggesting that macrophages do not contribute substantially in acute myocarditis [91]. The underlying explanation for such discrepant results still deserves further investigation, into, for instance, the level of disease inflammation, different macrophage subpopulations and differences in imaging techniques. However, the number of studies on cardiac inflammation remains relatively low. The optimal dose and most appropriate imaging time window await further evaluations in future studies. Once these fundamental questions are comprehensively addressed, FE-MRI, as an emerging and safe method for assessing inflammation, is anticipated to provide valuable clinical insights guiding interventions for cardiac inflammation.

#### Malignancy inflammation imaging

Once absorbed by inflammatory cells during the delayed phase, ferumoxytol acts as an intracellular CA, producing parenchymal enhancement of tumor tissue. FE-MRI has demonstrated additional advantages over GE-MRI in diagnosing malignant brain tumors, lymphomas, pancreatic cancer, osteosarcomas and gliomas. FE-MRI not only allows accurate assessment of rCBV in the blood pool phase but also provides inflammatory information in the delayed phase, hence increasing its attractiveness for MRI. It is known that GBCAs only enhance the ‘tip of the iceberg’ of the actual glioma area; FE-MRI during the delayed period can enhance a broader region of the brain tumor, thus providing additional insights compared to conventional GE-MRI [20]. As the paradigm in immunotherapy and precision oncology continues to evolve, the importance of FE-MRI's ability to capture inflammation has been further highlighted. Delayed T_1_-weighted imaging, up to 72 hours, can compare preoperative and postoperative tumor loads without a second contrast injection. FE-MRI enhances the precision of surgical biopsy targets, offers clearer delineation of pancreatic cancer boundaries compared to traditional structural imaging, identifies the presence or absence of enrichment in tumor-associated macrophages, and provides a quantitative imaging marker for macrophages in malignant gliomas [92]. This has significant implications for the clinical application of immune-targeted therapy stratification in tumor patients enriched in tumor-associated macrophages.

However, in assessing the inflammatory response during the delayed phase, FE-MRI faces several challenges. First, FE-MRI diagnostic findings may be confounded by hemorrhages, and other tissues that also present low signals on T_2_/T_2_*-weighted imaging. Second, the specificity of inflammation imaging with ferumoxytol is still limited. There exist some difficulties in distinguishing the source of inflammatory response; both brain tumors and inflammatory injuries can potentially produce similar inflammatory responses, making differentiation difficult.

### Mononuclear phagocyte system imaging

#### Ferumoxytol-enhanced MR lymphography

The ability of ferumoxytol to act as a CA for LNs opens up opportunities for imaging benign and malignant LNs throughout the body. The primary advantage of utilizing ferumoxytol-enhanced MR lymphography (FE-MRL) lies in its heightened sensitivity for pinpointing malignant LNs. This is because ferumoxytol provides deeper tissue insights than standard anatomical imaging. The rationale behind FE-MRL stems from the fact that malignant tumor cells replace the MPS in normal LNs. Consequently, after absorbing ferumoxytol, normal LNs exhibit reduced signal intensity on T_2_/T_2_*-weighted imaging, whereas malignant LNs, with their limited ferumoxytol uptake, display minimal signal alterations.

Since many primary malignancies spread through the lymphatic system, accurate diagnosis of LNs is crucial for tumor staging, treatment and prognosis [27]. FE-MRL has proven its effectiveness in identifying metastatic LNs in individuals with various cancers, such as prostate, bladder, kidney and sarcoma. Harisinghani and colleagues found comparable signal strengths in benign and malignant LNs pre-dose, with a significant signal reduction in benign nodes and minimal change in malignant ones post-dose [93]. Through sequential imaging, the authors determined that the optimal imaging time for assessing LNs is 24 hours after ferumoxytol administration. Moreover, they employed a 4.0 mg Fe/kg dosage, which is identical to that used in FE-MRA. This suggests that patients can conveniently undergo both FE-MRA and FE-MRL after a single injection. It was observed that FE-MRL demonstrated a high sensitivity rate of 98.0% in detecting metastatic LNs in patients with genitourinary tract cancers such as prostate, bladder and kidney [94].

FE-MRL has been shown to be a safe, non-invasive and accurate method for evaluating LNs, providing more information than conventional anatomical imaging. As a potential next-generation lymphotrophic CA, ferumoxytol has shown promise in LN characterization, yet its promising prospects necessitate validation in more extensive studies, especially concerning the technique's sensitivity and specificity.

#### Liver tumor heterogeneity and bone marrow lesions

Macrophages within the MPS can absorb ferumoxytol, suggesting that FE-MRI is a suitable tool for identifying liver and bone marrow abnormalities. Building on this premise, Lee et al. ingeniously utilized ferumoxytol's ability to accumulate and linger in Kupffer cells. They skillfully transformed what seemed to be a drawback into an advantage. Using low-dose FE-MRI, they achieved quantification of liver heterogeneity and distinct spatial separation of liver tumors from healthy liver tissue. This approach shows promise for guiding liver cancer radiotherapy [95]. Additionally, ferumoxytol significantly improved the detection of bone marrow metastasis using diffusion-weighted imaging [96], primarily owing to its unique pharmacokinetic properties that are aptly tailored for bone marrow imaging. Specifically, during the delayed phase, ferumoxytol is taken up by normal bone marrow but not by focal bone marrow lesions, offering a valuable complement to bone marrow imaging.

### Cell labeling and tracking

*In vivo* cell labeling and tracking are another important application of ferumoxytol as an intracellular CA, fundamental to the clinical translation of numerous biomedical applications, notably cell therapy. Owing to its excellent biocompatibility and powerful capability to monitor and predict the effects of cell therapy, FE-MRI has been preliminarily successful in clinical use [97]. The FE-MRI tracking of cells mainly involves two steps: first, labeling a certain number of cells to provide a signal detectable by MRI, and second, *in vivo* tracing to determine the distribution of labeled cells and their response to the environment. Various methods of ferumoxytol cell labeling have been proposed and are categorized into *ex vivo* and *in vivo* labeling. *In vivo* labeling utilizes the natural metabolic pathway, where ferumoxytol in the bloodstream is absorbed by the MPS, enters the bone marrow, and is then phagocytized by phagocytic cells. This process, for example, enables the isolation of labeled mesenchymal stem cells from the bone marrow [26]. Unlike *ex vivo* cell labeling, this *in vivo* approach avoids chemical or physical manipulation, potentially enhancing the clinical translation of cell therapy under stringent regulations. However, the *ex vivo* labeling method is more practical in terms of labeling a greater variety of cell types and quantities.

A critical diagnostic need in cell therapy is to assess the success of transplantation and to predict treatment outcomes early. FE-MRI has shown efficacy in tracking autologous bone marrow cell transplants in osteonecrosis patients, by monitoring cell quantity and distribution [93]. Notably, significantly fewer ferumoxytol-labeled cells were observed in a collapsed joint, hinting that early FE-MRI signal changes might forecast treatment results [97]. This was further explored in a minipig cartilage defect model, where persistent T_2_-weighted FE-MRI negative contrast signals at two weeks were correlated with better cartilage repair outcomes [98]. FE-MRI thus hastens the differentiation between successful and failed cell therapies, a process that normally takes months with conventional MRI. Another diagnostic priority in cell therapy is the detection of early transplant rejection reactions, a significant hurdle in treatment success. Intravenously injected ferumoxytol labels the macrophages infiltrating transplants, and FE-MRI has preclinically detected transplant rejection reactions [99]. However, caution is needed as macrophage enrichment due to injection trauma or non-rejection causes may lead to false positives.

It is important to recognize that there is currently no standardized imaging protocol for evaluating cell-containing implants, which are both varied and complex. Continuous enhancement of FE-MRI techniques and standards is crucial for effective clinical cell tracing. Typically, FE-MRI studies use T_2_- or T_2_*-weighted sequences for cell tracing. However, these sequences only offer qualitative estimates of transplanted cell numbers and are vulnerable to blooming artifacts and low-signal elements like air, which impact assessment accuracy and interpretability. Preliminary progress has been made in improving FE-MRI field strength and sequence optimization for more sensitive or quantitative tracing of ferumoxytol-labeled cells [100,101]. Despite this, challenges in specificity, such as *in vivo* labeling involving multiple cell types and macrophage infiltration around implants which generate false positive signals, complicate the interpretation of FE-MRI results. Utilizing co-localization with ferumoxytol and ^19^F labeling in multimodal MRI shows promise in overcoming these hurdles [102], but further safety assessments and clinical validation are needed.

## EMERGING IMAGING THEMES AND NEW TECHNICAL BREAKTHROUGHS

### Brain microvascular imaging

Diseases often originate on a microscopic scale and develop over time. Small vessel disease pertains to cerebral microvessel malfunctions, leading to manifestations like recent small subcortical infarcts and lacunes [103]. Undoubtedly, early monitoring and diagnosis of vascular alterations can enhance our understanding of disease origins and facilitate the advancement of therapeutic approaches. In clinical practice, MRA and CTA serve as reliable instruments for visualizing major arterial (ranging from M1 to M4) and venous (like dural sinus and internal cerebral veins) segments. These imaging techniques allow the visualization of vessels spanning roughly 250 μm to several millimeters, revealing merely the tip of the iceberg within the vast vascular network.

**Figure 7.**
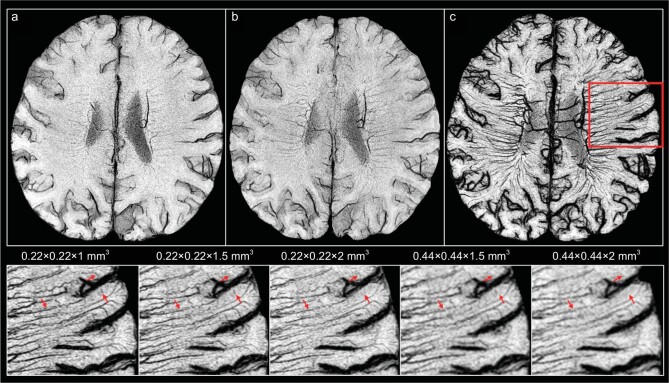
FE-SWI highlighting the prominence of brain microvessels (marked with red arrows). Pre-ferumoxytol SWI sequence images captured at (a) TE of 15 ms and (b) TE of 22.5 ms. (c) FE-SWI image post-administration of 4.0 mg/kg ferumoxytol. The last row illustrates FE-SWI at different resolutions, obtained by truncating elements in K-space. Reproduced from ref. [104].

Recently, FE-SWI has facilitated the visualization of human brain microvessels as diminutive as 50 μm by skillfully leveraging its blooming artifacts, opening up new opportunities for detecting and evaluating blood vessels at the microscopic level. This is accomplished through a gradual injection of ferumoxytol and by synergistically integrating multiple SWI data sets. Buch *et al.* employed FE-SWI in multiple sclerosis patients, observing an increased identification of vascular anomalies and a remarkable rise in microvessel density within lesioned regions [104] (Fig. 7). The team also used FE-SWI to delineate the microvascular mapping of the hippocampal. The imaging of brain microvessels holds significance in various neurodegenerative disorders, offering first-hand evidence illuminating the genesis and characteristics of such ailments. In future clinical pursuits, utilizing higher field strengths could potentially refine the resolution of FE-SWI or require lower doses. Presently, the described imaging technique needs around an hour for the examination of each patient and requires additional post-processing to obtain the final result. Faster imaging methods will further expand the clinical applicability of the technique.

### FE-MRI rapid imaging

There are three primary strategies for accelerated MR imaging: (i) parallel imaging that utilizes coil sensitivity profiles from multiple channels for image reconstruction; (ii) compressed sensing that reconstructs MRI images from under-sampled k-space data; and (iii) the emergent approach of deep-learning-based reconstruction. By combining parallel imaging with compressed sensing, the scan duration for 4D MUSIC cardiovascular FE-MRI was effectively halved, without sacrificing image clarity [60]. Zucker *et al.* recently crafted a deep-learning network for swift 2D cine CMR reconstruction, utilizing under-sampled k-space data and coil sensitivities [105]. This deep-learning approach notably speeds up acquisition times in ferumoxytol-enhanced cardiac cine imaging. Applying deep-learning solutions to fundamental image reconstruction processes provides a promising avenue for immediate clinical adoption. Given ferumoxytol's distinct benefits in CMR visuals, utilization of deep learning for FE-MRI image reconstruction could potentially introduce a groundbreaking approach to CMR.

Another aspect of FE-MRI rapid imaging is the development of a faster whole-body MRI protocol, specifically the ferumoxytol-enhanced whole-body MRA (FE-WB-MRA). Vascular diseases such as atherosclerosis exhibit pronounced systemic features, which often involve lesions in multiple vascular regions. This implies a need for WB-MRA for a comprehensive diagnostic outcome. The clinical value of WB-MRA is indisputable, especially in personalized medicine. However, the imaging speed and success rate of WB-MRA enhanced by GBCAs still require further improvement. Owing to the superior relaxation rate and extended blood half-life of ferumoxytol, FE-WB-MRA emerges as a particularly suitable method for WB-MRA, notably for the CKD demographic. In a study we are in the process of submitting, an FE-WB-MRA with a relatively low dose (3.0 mg/kg) showed a 100% success rate in a single attempt, offering an imaging window that lasts dozens of hours. Compared to GBCAs, FE-WB-MRA provides more pronounced and enduring vascular lumen enhancement. This suggests that faster imaging protocols with higher acceleration factors can be adopted to enhance time efficiency. Given the ease of administering ferumoxytol outside the examination room, and the GBCA method's need for synchronization and dual injections often spaced 10 minutes apart, FE-WB-MRA offers enhanced MRI efficiency, promoting wider clinical acceptance of WB-MRA.

### Multimodal image fusion and image segmentation

In clinical examinations, it is common to acquire images of multiple MR sequences. The multi-contrast capability of FE-MRI offers more comprehensive insights that are useful for diagnosis and therapeutic planning. Recent research showcases the potential of merging distinct FE-MRI sequences to gain novel perspectives into the analyzed regions. By merging fluid-attenuated inversion recovery (FLAIR) data with pre- and post-ferumoxytol SWI, a SWI-FLAIR fusion technique was created, achieving better micro-lesion detection compared to just FLAIR or SWI for vascular anomalies [104]. Combining calcification imaging from unenhanced CT with blood vessels from FE-MRA led to a detailed 3D fusion model ideal for comprehensive vascular planning [106].

**Figure 8.**
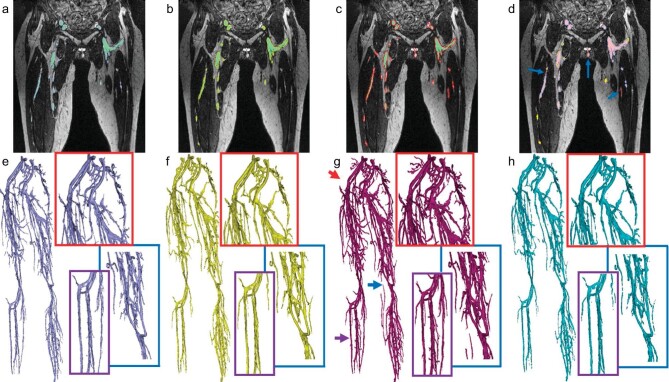
Representative results using an automatic segmentation algorithm to segment peripheral vessels from a high-resolution FE-MRA data set. (a–c) Planar views of segmentation results for 3D U-Net, 3D U-Net + DS and 3D U-Net + DS + AG, respectively, with contours marked in gray, yellow and red. (d) A comparison of the segmentation results for 3D U-Net, 3D U-Net + DS and 3D U-Net + DS + AG highlights the advantageous areas of 3D U-Net + DS + AG (blue arrows). (e–g) Volume renderings of segmentation results for 3D U-Net, 3D U-Net + DS and 3D U-Net + DS + AG, respectively. (h) Volume rendering of the manual segmentation result. The 3D U-Net + DS + AG yielded the best segmentation outcome (with the highest vascular density) and crucially identified some additional vessel branches that were overlooked during manual segmentation by radiologists (blue, red and purple arrows in (g)). DS = deep supervision. AG = attention gates. Reproduced with permission from ref. [41]. Copyright 2021, International Society for Magnetic Resonance in Medicine.

Previous studies have demonstrated that FE-MRA can provide uniform enhancement in arteries and veins during its blood pool phase. For a swift and efficient interpretation of FE-MRA images, differentiating and segmenting arteries from veins becomes crucial. Ghodrati *et al.* developed a unique two-stage deep-learning approach to achieve precise segmentation of peripheral arteries and veins in FE-MRA [41] (Fig. 8). The imaging during the initial phase employed an attention-gated 3D U-Net for blood vessel extraction, followed by a semi-automatic region-growing algorithm in the subsequent phase to further differentiate artery-vein. Both quantitative and qualitative outcomes highlighted the pipeline's ability to segment small vessels, which even expert radiologists might miss, in a significantly reduced time (i.e. in a matter of minutes rather than hours).

## FUTURE DIRECTIONS

Based on the current applications and advantages of ferumoxytol described earlier, and drawing on our experience with ferumoxytol synthesis and insights from ongoing clinical trials, we envision a future in which FE-MRI prioritizes patient-specific care, quantitative MRI imaging and molecular imaging.

### Patient-specific care

First, ferumoxytol offers multiple advantages, including a proven safety profile, a one-stop-shop protocol for high-resolution imaging of multiple vascular beds, and intravenous iron supplementation, which all boost the forward progress of patient-specific care. Given that vascular diseases often affect multiple vascular beds [107], utilizing ferumoxytol for one-stop whole-body multivessel bed assessment in patients with coexisting vascular disease is extremely beneficial, especially for patients with diabetes and cardiovascular disease. As the venous system has garnered increased attention in recent years, a more comprehensive assessment of multiple vessel beds will provide more thorough diagnostic information in various cases. In clinical practice, it is technically feasible to achieve 3D modeling of patient-specific vascular anatomy based on FE-MRI data. The combination of 3D modeling and hemodynamic analysis is highly advantageous in several applications such as preoperative surgical planning [76]. In the temporal dimension, the intravascular half-life is ∼14.5 hours [17]. The longer imaging time window opens up new opportunities for continuous preoperative and postoperative evaluations for surgery. Furthermore, more than 1.2 billion people worldwide suffer from iron deficiency anemia [108], making iron deficiency a prevalent clinical issue. This confers a dual function to ferumoxytol for FE-MRI and iron supplementation. Thus, we anticipate that in the future, with the continued advancement of automatic segmentation technology and 3D printing technology, FE-MRI will move toward patient-specific care especially for a wide range of special groups, including but not limited to: CKD, maternal and pediatric patients; people with previous GBCA allergies; and people with claustrophobia.

### Innovative FE-MRI technology

Given its unique dual-modality contrast and extended intravascular half-life, new pulse sequences tailored for ferumoxytol can be developed. This will enable the integration of accelerated imaging techniques and quantitative methods, achieving both rapid and quantitative imaging. For example, integrated assessment of cardiac structural imaging and molecular imaging can be conducted in the same sequence. Compared to GE-MRI, FE-MRI is particularly suitable for CMR and highly promising for clinical applications in the future after integrating the assessment of myocardial activity, myocardial inflammation, etc. Ferumoxytol stays within the blood pool for a considerable period of time, thus paving the way for the fast acquisition of multiple contrast (e.g. T_1_, T_2_*, SWI) signals for FE-MRI. Furthermore, quantitative imaging may also be an important direction for FE-MRI technology. While quantitative susceptibility mapping can already quantify the iron content in blood or in ferumoxytol-labeled cells, it still faces challenges, such as extended scanning times and intricate post-processing procedures. It is anticipated that multiple quantitative iron imaging methods at more sites will be further accelerated. Furthermore, the synergistic use of GBCAs and ferumoxytol has been initially proven to offer complementary diagnostic benefits, such as improving the detection rate of liver metastases in colorectal cancer [109]. The joint utilization of both may give rise to a series of novel clinical applications and image analysis methods.

### Expanding molecular imaging applications

Ferumoxytol's unique structure and straightforward cell-labeling capability make it well-suited for molecular and cellular imaging. The nano-iron core of ferumoxytol, which contains thousands of iron atoms, exhibits a strong T_2_* effect, while its polydextrose sorbitol carboxymethyl ether coating allows linkage to many targeted molecules and therapeutic drugs. These properties further enhance the sensitivity of MRI, opening up a broad spectrum of possibilities for integrated targeted monitoring, assessment and treatment in the realm of cell therapy. Ferumoxytol has demonstrated high sensitivity and specificity in labeling cells for MRI tracking and in the imaging of active macrophages. It is anticipated that targeted imaging and tailored therapy monitoring will be expanded in future applications. Additionally, ferumoxytol has been proven as an apt platform for hybridized PET tracers [110]. In this sense, the development of a multimodal imaging method based on PET/MRI could offer fresh opportunities for molecular imaging applications using ferumoxytol.

## CONCLUSIONS

Despite its off-label status, FE-MRI has undeniably transformed CE-MRI clinical practice over the last decade, particularly for CKD patients as well as maternal and pediatric populations where the use of GBCAs is not advisable. Here, we summarize the clinical applications and emerging themes of FE-MRI, offering our insights into its prospective advancements.

Ferumoxytol, currently the most utilized gadolinium-free alternative, offers multidimensional advantages over GBCAs, which position it as a leading candidate in the next generation of MRI CAs. Distinguished within the SPIO family for nanomedical imaging, ferumoxytol's clinical success introduces an additional facet to non-invasive disease diagnosis and provides a crucial reference for developing and clinically translating new MRI CAs. Firstly, it demonstrates higher relaxivity compared to first-generation GBCAs, effectively reducing the required CA dosage. Secondly, its distinctive pharmacokinetics facilitate comprehensive anatomical, functional and molecular imaging, a notable capability that is markedly absent in current GBCAs. In particular, its extended blood pool half-life of a dozen or so hours ensures robust and efficient FE-WB-MRA, seamlessly enabling high-resolution MRA in specific groups, such as children, thereby broadening its applicability. Thirdly, although part of the SPIO family, ferumoxytol reveals balanced bimodal imaging potential through its ingeniously designed nanostructure, a departure from the traditional role of SPIOs as primarily T_2_ CAs. Fourthly, its lack of nephrotoxicity addresses a crucial need in MRI CAs for patients with CKD and other renal conditions.

In conclusion, the unique pharmacokinetic and MRI properties of ferumoxytol determine its specific range of applications. With the increasing disclosure of safety data and the innovation of new methods, the clinical application and exploration of FE-MRI across various indications are expected to expand further in the future. Additionally, the advent of a novel generic form of ferumoxytol, exhibiting comparable relaxometry and *in vivo* MRI characteristics, suggests the increased accessibility of this CA [111]. It is plausible to expect that ongoing research and technological innovation will reveal a plethora of new clinical uses beyond the scope of traditional GE-MRI. Moreover, the progression and implementation of advanced computer post-processing techniques are likely to accelerate this evolutionary process.

## Supplementary Material

nwae057_Supplemental_File
