# Correction to: Unveiling the next generation of MRI contrast agents: current insights and perspectives on ferumoxytol-enhanced MRI

**DOI:** 10.1093/nsr/nwae156

**Published:** 2024-05-20

**Authors:** Guangxiang Si, Yue Du, Peng Tang, Gao Ma, Zhaochen Jia, Xiaoyue Zhou, Dan Mu, Yan Shen, Yi Lu, Yu Mao, Chuan Chen, Yan Li, Ning Gu

**Affiliations:** Jiangsu Key Laboratory for Biomaterials and Devices, School of Biological Science and Medical Engineering, Southeast University, Nanjing 210009, China; Key Laboratory for Bio-Electromagnetic Environment and Advanced Medical Theranostics, School of Biomedical Engineering and Informatics, Nanjing Medical University, Nanjing 211166, China; Key Laboratory for Bio-Electromagnetic Environment and Advanced Medical Theranostics, School of Biomedical Engineering and Informatics, Nanjing Medical University, Nanjing 211166, China; Department of Radiology, the First Affiliated Hospital of Nanjing Medical University, Nanjing 210029, China; Jiangsu Key Laboratory for Biomaterials and Devices, School of Biological Science and Medical Engineering, Southeast University, Nanjing 210009, China; MR Collaboration, Siemens Healthineers Ltd., Shanghai 200126, China; Department of Radiology, Affiliated Nanjing Drum Tower Hospital of Nanjing University Medical School, Nanjing 210008, China; Key Laboratory for Bio-Electromagnetic Environment and Advanced Medical Theranostics, School of Biomedical Engineering and Informatics, Nanjing Medical University, Nanjing 211166, China; School of Mathematical Sciences, Capital Normal University, Beijing 100048, China; Nanjing Key Laboratory for Cardiovascular Information and Health Engineering Medicine, Institute of Clinical Medicine, Nanjing Drum Tower Hospital, Medical School, Nanjing University, Nanjing 210093, China; Jiangsu Key Laboratory for Biomaterials and Devices, School of Biological Science and Medical Engineering, Southeast University, Nanjing 210009, China; Jiangsu Key Laboratory for Biomaterials and Devices, School of Biological Science and Medical Engineering, Southeast University, Nanjing 210009, China; Nanjing Key Laboratory for Cardiovascular Information and Health Engineering Medicine, Institute of Clinical Medicine, Nanjing Drum Tower Hospital, Medical School, Nanjing University, Nanjing 210093, China; Jiangsu Key Laboratory for Biomaterials and Devices, School of Biological Science and Medical Engineering, Southeast University, Nanjing 210009, China

In Table 1 of the original article titled ‘Unveiling the next generation of MRI contrast agents: current insights and perspectives on ferumoxytol-enhanced MRI’ (*National Science Review*, Volume 11, Issue 5, May 2024, nwae057, https://doi.org/10.1093/nsr/nwae057), there was an inadvertent error in the provided trade names and market status information of some contrast agents. The corrected version of Table 1 is presented below.

Additionally, the second sentence of the second paragraph in Page 2, which originally stated ‘…, holds the unique position as the only iron-based CA approved for clinical intravenous use.’, should be revised to read ‘…, occupies a distinctive role due to its versatility in imaging various bodily systems and its widespread clinical indications.’ Another sentence within the same paragraph previously read ‘Moreover, the recent global discontinuation of Omniscan (gadodiamide), one of the GBCAs, …’ should be corrected to ‘Moreover, the recent discontinuation of Omniscan (gadodiamide) in Canada, one of the GBCAs, …’. Following the corrections above, it should be noted that the postal code for the second institution has been updated.

**Table 1.** Clinically approved intravenous MRI contrast agents.

Contrast agent category	Trade name	Generic name	Distribution type in body	Indications	Current market status
**Gadolinium-based contrast agents**	Dotarem, Clariscan	Gadoterate meglumine	Extracellular fluid agent	CNS^a^	Marketed
	ProHance	Gadoteridol		CNS, head-neck MRI^b^	Marketed
	Gadovist, Gadavist	Gadobutrol		CNS, breast MRI, MRA^c^, Cardiac MRI	Marketed
	Magnevist	Gadopentetate dimeglumine		CNS, MRA	Marketed
	Omniscan	Gadodiamide		CNS, MRA	Marketed
	OptiMARK	Gadoversetamide		CNS, liver MRI	Marketed​
	MultiHance	Gadobenate dimeglumine		CNS, MRA	Marketed​
	Vasovist, Ablavar	Gadofosveset trisodium	Blood pool agent	MRA	Discontinued (2017)
	Primovist, Eovist	Gadoxetate disodium, Gadoxetic acid	Targeted/organ-specific agent	Liver MRI	Marketed​
**Iron-based contrast agents**	Feraheme, Rienso	Ferumoxytol	Blood pool agent, targeted/organ-specific agent	IDA^d^, MRI (off-label)	Marketed​​
	Feridex, Endorem	Ferumoxides	Targeted/organ-specific agent	Liver MRI	Discontinued (2008)
	Resovist, Cliavist, Resotran	Ferucarbotran	Targeted/organ-specific agent	Liver MRI	Marketed​
**Manganese-based contrast agents**	Teslascan	Mangafodipir trisodium	Targeted/organ-specific agent	Liver and pancreas MRI	Discontinued (2012)

a CNS, central nervous system; ^b^MRI, magnetic resonance imaging; ^c^MRA, magnetic resonance angiography; ^d^IDA, iron deficiency anemia.

