# Supplementary material for: Unveiling the next generation of MRI contrast agents: current insights and perspectives on ferumoxytol-enhanced MRI
Source: Natl Sci Rev. 2024 Feb 7;11(5):nwae057. doi: 10.1093/nsr/nwae057 (PMC10989670; doi:10.1093/nsr/nwae057)
Supplement: nwae057_Supplemental_File [file nwae057_supplemental_file.pdf]

Summary of ferumoxytol-enhanced MRI Clinical Research Articles

Accompanying the review article titled:
"Unveiling the next generation of MRI contrast agents: Current insights and perspectives on ferumoxytol-enhanced MRI"
This Excel spreadsheet provides a comprehensive aggregation of clinical research articles focusing on ferumoxytol-enhanced MRI. The articles have been systematically categorized based on their specific clinical indications. Our intention is to offer readers a consolidated reference point, aiding in a deeper understanding of the subject matter discussed in our review. We believe this structured summary will facilitate easy access to pertinent studies and streamline the process of referencing specific research in the context of ferumoxytol-enhanced MRI clinical applications.

Clinical Indication			Year	Author(s)	Sample Size	Title
Blood Pool Phase	Arterial System	Peripheral Artery Disease	2005	Li, Wei, et al.	12	First-pass contrast-enhanced magnetic resonance angiography in humans using ferumoxytol, a novel ultrasmall superparamagnetic iron oxide (USPIO)-based blood pool agent.
			2015	Walker, Joy P., et al.	10	Ferumoxytol-enhanced magnetic resonance angiography is a feasible method for the clinical evaluation of lower extremity arterial disease.
			2015	Hope, Michael D., et al.	102	Vascular Imaging With Ferumoxytol as a Contrast Agent.
		Transcatheter Aortic Valve Replacement	2017	Kallianos, Kimberly, et al.	7	Ferumoxytol MRA for transcatheter aortic valve replacement planning with renal insufficiency.
			2018	Zhu, Chengcheng, et al.	23	Gated thoracic magnetic resonance angiography at 3T: noncontrast versus blood pool contrast.
			2018	Nguyen, Kim-Lien, et al.	26	Ferumoxytol-enhanced MR Angiography for Vascular Access Mapping before Transcatheter Aortic Valve Replacement in Patients with Renal Impairment: A Step Toward Patient-specific Care.
			2022	Melo, Diana, et al.	31	Accuracy of Ferumoxytol-Enhanced MRA-Guided TAVR in Patients With Chronic Kidney Disease.
		Pulmonary Artery and Pulmonary Embolism	2003	Prince, Martin R., et al.	5	A pilot investigation of new superparamagnetic iron oxide (ferumoxytol) as a contrast agent for cardiovascular MRI
			2015	Ruangwattanapaisarn, N., et al.	23	Ferumoxytol as an off-label contrast agent in body 3T MR angiography: a pilot study in children.
			2015	Hope, Michael D., et al.	8	Vascular Imaging With Ferumoxytol as a Contrast Agent.
			2019	Knobloch, Gesine, et al.	20	Comparison of gadolinium-enhanced and ferumoxytol-enhanced conventional and UTE-MRA for the depiction of the pulmonary vasculature.
			2020	Aghayev, Ayaz, et al.	2	Alternative Diagnostic Strategy for the Assessment and Treatment of Pulmonary Embolus: A Case Series.
			2023	Starekova, Jitka, et al.	94	Pulmonary MRA During Pregnancy: Early Experience With Ferumoxytol.
		Relative Cerebral Blood Volume and Functional Magnetic Resonance Imaging	2011	Gahramanov, Seymur, et al.	14	Potential for differentiation of pseudoprogression from true tumor progression with dynamic susceptibility-weighted contrast-enhanced magnetic resonance imaging using ferumoxytol vs. gadoteridol: a pilot study.
			2012	Thompson, Eric M., et al.	7	Dual contrast perfusion MRI in a single imaging session for assessment of pediatric brain tumors.
			2012	Qiu, Deqiang, et al.	4	Contrast-enhanced functional blood volume imaging (CE-fBVI): enhanced sensitivity for brain activation in humans using the ultrasmall superparamagnetic iron oxide agent ferumoxytol.
			2013	Christen, Thomas, et al.	7	High-resolution cerebral blood volume imaging in humans using the blood pool contrast agent ferumoxytol.
			2013	Varallyay, Csanad G., et al.	65	High-resolution steady-state cerebral blood volume maps in patients with central nervous system neoplasms using ferumoxytol, a superparamagnetic iron oxide nanoparticle.
			2013	Gahramanov, Seymur, et al.	19	Pseudoprogression of Glioblastoma after Chemo- and Radiation Therapy: Diagnosis by Using Dynamic Susceptibility-weighted Contrast-enhanced Perfusion MR Imaging with Ferumoxytol versus Gadoteridol and Correlation with Survival.
			2013	Farrell, Brian T., et al.	20	Using iron oxide nanoparticles to diagnose CNS inflammatory diseases and PCNSL.
			2013	D'Arceuil, Helen, et al.	8	Ferumoxytol enhanced resting state fMRI and relative cerebral blood volume mapping in normal human brain.
			2014	Nasseri, Morad, et al.	56	Evaluation of pseudoprogression in patients with glioblastoma multiforme using dynamic magnetic resonance imaging with ferumoxytol calls RANO criteria into question.
			2016	Netto, Joao Prola, et al.	16	Misleading early blood volume changes obtained using ferumoxytol-based magnetic resonance imaging perfusion in high grade glial neoplasms treated with bevacizumab
			2017	Li, Xin, et al.	17	Pseudo-extravasation rate constant of dynamic susceptibility contrast-MRI determined from pharmacokinetic first principles.
			2018	de Zwart, Jacco A., et al.	5	Impulse response timing differences in BOLD and CBV weighted fMRI.
			2018	Maralani, Pejman Jabehdar, et al.	15	Hypoxia Detection in Infiltrative Astrocytoma: Ferumoxytol-based Quantitative BOLD MRI with Intraoperative and Histologic Validation.
			2018	Rivera-Rivera, Leonardo A., et al.	20	Comparison of ferumoxytol-based cerebral blood volume estimates using quantitative R(1) and R2* relaxometry.
			2018	Varallyay, Csanad G., et al.	54	Cerebral blood volume mapping with ferumoxytol in dynamic susceptibility contrast perfusion MRI: Comparison to standard of care.
			2019	Rivera-Rivera, Leonardo A., et al.	19	Measurements of cerebral blood volume using quantitative susceptibility mapping, R(2) * relaxometry, and ferumoxytol-enhanced MRI.
			2019	Thrippleton, Michael J., et al.	12	MRI Relaxometry for Quantitative Analysis of USPIO Uptake in Cerebral Small Vessel Disease.
		Congenital Heart Disease Evaluation	2016	Hanneman, Kate, et al.	22	Assessment of the precision and reproducibility of ventricular volume, function, and mass measurements with ferumoxytol-enhanced 4D flow MRI.
			2016	Cheng, Joseph Y., et al.	23	Comprehensive Motion-Compensated Highly Accelerated 4D Flow MRI With Ferumoxytol Enhancement for Pediatric Congenital Heart Disease.
			2017	Nguyen, Kim-Lien, et al.	40	4D MUSIC CMR: value-based imaging of neonates and infants with congenital heart disease.
			2017	Zhou, Ziwu, et al.	13	Accelerated ferumoxytol-enhanced 4D multiphase, steady-state imaging with contrast enhancement (MUSIC) cardiovascular MRI: validation in pediatric congenital heart disease.
			2017	Han, Fei, et al.	10	Self-gated 4D multiphase, steady-state imaging with contrast enhancement (MUSIC) using rotating cartesian K-space (ROCK): Validation in children with congenital heart disease.
			2017	Nguyen, Kim-Lien, et al.	340	Ferumoxytol enhanced black-blood cardiovascular magnetic resonance imaging.
			2017	Lai, Lillian M., et al.	21	Feasibility of ferumoxytol-enhanced neonatal and young infant cardiac MRI without general anesthesia.
			2018	Zhou, Ziwu, et al.	12	Improved 4D cardiac functional assessment for pediatric patients using motion-weighted image reconstruction.
			2018	Mukai, Kanae, et al.	10	4D flow image quality with blood pool contrast: a comparison of gadofosveset trisodium and ferumoxytol.
			2018	Wise-Faberowski, Lisa, et al.	61	Safety of ferumoxytol in children undergoing cardiac MRI under general anaesthesia.
			2021	Nguyen, Kim-Lien, et al.	206	Four-dimensional Multiphase Steady-State MRI with Ferumoxytol Enhancement: Early Multicenter Feasibility in Pediatric Congenital Heart Disease.
			2022	Yoshida, Takegawa, et al.	50	Ferumoxytol-enhanced 4D multiphase, steady-state imaging with magnetic resonance in congenital heart disease: ventricular volume and function across 2D and 3D software platforms.
			2022	Kollar, Sarah E., et al.	21	Impact of ferumoxytol vs gadolinium on 4D flow cardiovascular magnetic resonance measurements in small children with congenital heart disease.
		Coronary Magnetic Resonance Angiography	2018	Chin, Matthew S., et al.	5	Intraluminal Assessment of Coronary Arteries With Ferumoxytol-Enhanced Magnetic Resonance Angiography.
			2021	Miller, Timothy, et al.	13	Ferumoxytol-Enhanced Coronary Magnetic Resonance Angiography Compared to Invasive Coronary Angiography for Detection of Epicardial Coronary Artery Disease.
			2021	Heerfordt, John, et al.	6	Similarity-driven multi-dimensional binning algorithm (SIMBA) for free-running motion-suppressed whole-heart MRA.
			2022	Roy, Christopher W., et al.	18	Free-running cardiac and respiratory motion-resolved 5D whole-heart coronary cardiovascular magnetic resonance angiography in pediatric cardiac patients using ferumoxytol.
			2023	Rezkalla, J., et al.	1	Right ventricle-dependent coronary circulation diagnosed by non-invasive ferumoxytol-enhanced 4D cardiac magnetic resonance angiography in pulmonary atresia with intact ventricular septum
			2023	Dong, Zhou, et al.	30	Diagnostic Performance and Safety of a Novel Ferumoxytol-Enhanced Coronary Magnetic Resonance Angiography.
	Venous System	Deep Venous Thrombosis, Venous Evaluation in Pediatric Patients, Preoperative Analysis of Deep Inferior Epigastric Artery Perforator Flap, and Central Venous Stenosis and Occlusion	2014	Bashir, Mustafa R., et al.	34	Retrospective Assessment of the Utility of an Iron-Based Agent for Contrast-Enhanced Magnetic Resonance Venography In Patients With Endstage Renal Diseases.
			2016	Luhar, Aarti, et al.	20	Contrast-enhanced magnetic resonance venography in pediatric patients with chronic kidney disease: initial experience with ferumoxytol.
			2019	Shahrouki, Puja, et al.	52	High resolution, 3-dimensional Ferumoxytol-enhanced cardiovascular magnetic resonance venography in central venous occlusion.
			2020	Gallo, Christopher JR, et al.	35	Ferumoxytol-enhanced MR Venography of the Central Veins of the Thorax for the Evaluation of Stenosis and Occlusion in Patients with Renal Impairment.
			2021	Dortch, John, et al.	59	Preoperative Analysis of Venous Anatomy Before Deep Inferior Epigastric Perforator Free-Flap Breast Reconstruction Using Ferumoxytol-enhanced Magnetic Resonance Angiography.
			2022	Shahrouki, Puja, et al.	20	High-resolution threedimensional contrastenhanced magnetic resonance venography in children: comparison of gadofosveset trisodium with ferumoxytol.
		Arteriovenous Fistula Surgery Planning and Routine Monitoring	2012	Sigovan, Monica, et al.	10	USPIO-enhanced MR Angiography of Arteriovenous Fistulas in Patients with Renal Failure.
			2020	Stoumpos, Sokratis, et al.	59	Ferumoxytol MR Angiography versus Duplex US for Vascular Mapping before Arteriovenous Fistula Surgery for Hemodialysis.
			2022	Hyde-Linaker, George, et al.	1	Patient-specific computational haemodynamics associated with the surgical creation of an arteriovenous fistula.

	Combined Arteriovenous Evaluation	Arteriovenous Malformations	2011	Dósa, Edit, et al.	19	MRI Using Ferumoxytol Improves the Visualization of Central Nervous System Vascular Malformations.
			2018	Iv, Michael, et al.	21	High-resolution 3D volumetric contrast-enhanced MR angiography with a blood pool agent (ferumoxytol) for diagnostic evaluation of pediatric brain arteriovenous malformations.
			2019	Huang, Yuhao, et al.	15	Ferumoxytol-enhanced MRI for surveillance of pediatric cerebral arteriovenous malformations.
			2020	Khan, Sarah N., et al.	10	Comparison of Ferumoxytol-enhanced MR Angiography and CT Angiography for the Detection of Pulmonary Arteriovenous Malformations in Hereditary Hemorrhagic Telangiectasia: Initial Results."
		Allograft Vasculature Assessment	2013	Bashir, Mustafa R., et al.	16	Renal transplant imaging using magnetic resonance angiography with a nonnephrotoxic contrast agent.
			2016	Corwin, M. T., et al.	15	MR angiography of renal transplant vasculature with ferumoxytol:: comparison of high-resolution steady-state and first-pass acquisitions
			2017	Fananapazir, Ghaneh, et al.	33	Comparison of ferumoxytol-enhanced MRA with conventional angiography for assessment of severity of transplant renal artery stenosis.
			2018	Stoumpos, Sokratis, et al.	20	Ferumoxytol-enhanced magnetic resonance angiography for the assessment of potential kidney transplant recipients.
			2020	Stoumpos, Sokratis, et al.	36	Ferumoxytol MR Angiography: A Novel Technique for Assessing Iliac Vasculature in Potential Kidney Transplant Recipients.
			2021	Aghayev, Ayaz, et al.	1	Transplant renal artery and vein occlusion evaluated with ferumoxytol-enhanced magnetic resonance angiography.
Delayed Phase	Inflammatory Imaging	Vascular System Inflammation Imaging	2012	Hasan, David M., et al.	11	Macrophage imaging within human cerebral aneurysms wall using ferumoxytol-enhanced MRI: a pilot study.
			2012	Hasan, David M., et al.	22	Early change in ferumoxytol-enhanced magnetic resonance imaging signal suggests unstable human cerebral aneurysm: a pilot study.
			2012	Hasan, David M., et al.	4	Ferumoxytol-enhanced MRI to Image Inflammation within Human Brain Arteriovenous Malformations: A Pilot Investigation.
			2013	Hasan, David M., et al.	5	Imaging aspirin effect on macrophages in the wall of human cerebral aneurysms using ferumoxytol-enhanced MRI: preliminary results.
			2013	Hasan, David M., et al.	11	Evidence that acetylsalicylic acid attenuates inflammation in the walls of human cerebral aneurysms: preliminary results.
			2017	Smits, Loek P., et al.	9	Evaluation of ultrasmall superparamagnetic iron-oxide (USPIO) enhanced MRI with ferumoxytol to quantify arterial wall inflammation.
			2017	Smits, Loek P., et al.	9	Evaluation of ultrasmall superparamagnetic iron-oxide (USPIO) enhanced MRI with ferumoxytol to quantify arterial wall inflammation.
			2018	Hedgire, Sandeep, et al.	16	Ultrasmall superparamagnetic iron oxide nanoparticle uptake as noninvasive marker of aortic wall inflammation on MRI: proof of concept study.
			2020	Usman, Ammara, et al.	10	Ferumoxytol-enhanced three-dimensional magnetic resonance imaging of carotid atheroma- a feasibility and temporal dependence study.
			2020	Langsjoen, Jens, et al.	15	A comparison of ferumoxytol with gadolinium as contrast agents for the diagnostic magnetic resonance imaging of osteomyelitis.
		Cardiac Inflammation Imaging	2012	Alam, Shirjel R., et al.	16	Ultrasmall superparamagnetic particles of iron oxide in patients with acute myocardial infarction: early clinical experience.
			2013	Yilmaz, Ali, et al.	14	Imaging of myocardial infarction using ultrasmall superparamagnetic iron oxide nanoparticles: a human study using a multi-parametric cardiovascular magnetic resonance imaging approach.
			2017	Stirrat, Colin G., et al.	31	Ferumoxytol-enhanced magnetic resonance imaging assessing inflammation after myocardial infarction.
			2018	Stirrat, Colin G., et al.	24	Ferumoxytol-enhanced magnetic resonance imaging in acute myocarditis.
			2019	Stirrat, Colin G., et al.	21	Ferumoxytol-enhanced MRI in patients with prior cardiac transplantation.
		Malignancy Inflammation Imaging	2007	Neuwelt, Edward A., et al.	12	The potential of ferumoxytol nanoparticle magnetic resonance imaging, perfusion, and angiography in central nervous system malignancy: a pilot study.
			2011	Dósa, Edit, et al.	26	Magnetic resonance imaging of intracranial tumors: intra-patient comparison of gadoteridol and ferumoxytol.
			2013	Farrell, Brian T., et al.	20	Using iron oxide nanoparticles to diagnose CNS inflammatory diseases and PCNSL.
			2014	Hedgire, Sandeep S., et al.	8	Enhanced primary tumor delineation in pancreatic adenocarcinoma using ultrasmall super paramagnetic iron oxide nanoparticle-ferumoxytol: an initial experience with histopathologic correlation.
			2018	Aghighi, Maryam, et al.	25	Magnetic Resonance Imaging of Tumor-Associated Macrophages: Clinical Translation.
			2019	Iv, Michael, et al.	10	Quantification of Macrophages in High-Grade Gliomas by Using Ferumoxytol-enhanced MRI: A Pilot Study.
			2020	Siedek, Florian, et al.	22	Comparison of ferumoxytol- and gadolinium chelate-enhanced MRI for assessment of sarcomas in children and adolescents.
	Mononuclear Phagocyte System Imaging	Ferumoxytol-enhanced MR lymphography	2007	Harisinghani, Mukesh, et al.	10	Utility of a new bolus-injectable nanoparticle for clinical cancer staging.
			2013	McDermott, Shaunagh, et al.	13	Accurate prediction of nodal status in preoperative patients with pancreatic ductal adenocarcinoma using next-gen nanoparticle.
			2015	Turkbey, Baris, et al.	15	A Phase I Dosing Study of Ferumoxytol for MR Lymphography at 3 T in Patients With Prostate Cancer.
			2020	Wells, Shane A., et al.	12	Pharmacokinetics of Ferumoxytol in the Abdomen and Pelvis: A Dosing Study with 1.5- and 3.0-T MRI Relaxometry.
			2020	Turkbey, Baris, et al.	39	Ferumoxytol-Enhanced MR Lymphography for Detection of Metastatic Lymph Nodes in Genitourinary Malignancies: A Prospective Study.
			2020	Muehe, Anne Monika, et al.	42	Differentiation of benign and malignant lymph nodes in pediatric patients on ferumoxytol-enhanced PET/MRI.
		Liver Tumors Heterogeneity and Bone Marrow Lesions	2022	Lee, D., et al.	12	Quantifying Liver Heterogeneity via R2*-MRI with Super-Paramagnetic Iron Oxide Nanoparticles (SPION) to Characterize Liver Function and Tumor.
			2023	Rashidi, Ali, et al.	26	Improved Detection of Bone Metastases in Children and Young Adults with Ferumoxytol-enhanced MRI.
	Cell Labeling and Tracking		2018	Theruvath, Ashok J et al.	4	Tracking Cell Transplants in Femoral Osteonecrosis with Magnetic Resonance Imaging: A Proof-of-Concept Study in Patients
Emerging Imaging Themes and New Technical Breakthroughs	Brain microvascular imaging		2018	Liu, Saifeng, et al.	3	Susceptibility weighted imaging and quantitative susceptibility mapping of the cerebral vasculature using ferumoxytol.
			2020	Buch, Sagar, et al.	13	Subvoxel vascular imaging of the midbrain using USPIO-Enhanced MRI.
			2021	Buch, Sagar, et al.	20	Revealing vascular abnormalities and measuring small vessel density in multiple sclerosis lesions using USPIO.
			2022	Buch, Sagar, et al.	39	Vascular mapping of the human hippocampus using Ferumoxytol-enhanced MRI.
	FE-MRI Rapid Imaging		2017	Zhou, Ziwu, et al.	13	Accelerated ferumoxytol-enhanced 4D multiphase, steady-state imaging with contrast enhancement (MUSIC) cardiovascular MRI: validation in pediatric congenital heart disease.
			2021	Zucker, Evan J., et al.	22	Free-breathing Accelerated Cardiac MRI Using Deep Learning: Validation in Children and Young Adults.
	Multi-Modal Image Fusion and Image Segmentation		2015	Gaglia, Jason L., et al.	11	Noninvasive mapping of pancreatic inflammation in recent-onset type-1 diabetes patients.
			2021	Buch, Sagar, et al.	20	Revealing vascular abnormalities and measuring small vessel density in multiple sclerosis lesions using USPIO.
			2020	Yoshida, Takegawa, et al.	15	Intermodality feature fusion combining unenhanced computed tomography and ferumoxytol-enhanced magnetic resonance angiography for patient-specific vascular mapping in renal impairment.
			2017	Schwein, Adeline, et al.	10	Feasibility of three-dimensional magnetic resonance angiography-fluoroscopy image fusion technique in guiding complex endovascular aortic procedures in patients with renal insufficiency.
			2022	Ghodrati, Vahid, et al.	45	Automatic segmentation of peripheral arteries and veins in ferumoxytol-enhanced MR angiography.
